# Laparoscopic Myomectomy and Pregnancy Outcomes: A Narrative Review

**DOI:** 10.7759/cureus.87268

**Published:** 2025-07-04

**Authors:** Vaia G Sarli, Aikaterini Lydia Vogiatzoglou, Theofanakis Charalampos, Dimos Sioutis, Periklis Panagopoulos, Nikolaos Machairiotis

**Affiliations:** 1 Third Department of Obstetrics and Gynecology, Attikon University Hospital, National and Kapodistrian University of Athens, Athens, GRC

**Keywords:** fibroids, infertility, laparoscopy, myomectomy, pregnancy outcome, reproductive surgery

## Abstract

Uterine fibroids (leiomyomas) are common benign tumors in women of reproductive age and are often associated with infertility, heavy menstrual bleeding, and pelvic discomfort. Laparoscopic myomectomy (LSM) has emerged as a fertility-preserving alternative to open surgery, offering faster recovery and reduced perioperative morbidity. This review aims to evaluate the effectiveness of laparoscopic myomectomy in improving pregnancy outcomes among women with fibroid-related infertility. A literature review was conducted using PubMed and Web of Science to identify retrospective cohort studies, case reports, and systematic reviews assessing fertility outcomes after LSM. Data on patient demographics, fibroid characteristics, surgical technique, and reproductive outcomes were extracted and analyzed to assess trends in pregnancy rates, delivery methods, and complications. Fifteen retrospective cohort studies and one case report were included in the analysis. Postoperative pregnancy rates ranged from 44% to 62% across studies, with a high proportion of successful full-term deliveries. Vaginal delivery was feasible in many cases, and complications such as uterine rupture were rare (<1%). Proper suturing techniques and minimally invasive approaches contributed to favorable reproductive outcomes, with LSM proving to be effective even in cases of multiple or large fibroids. Laparoscopic myomectomy is a safe and effective surgical option for women seeking fertility preservation. The procedure is associated with high postoperative pregnancy and live birth rates, minimal complications, and promising obstetric outcomes. Continued advancements in laparoscopic techniques and further large-scale studies with long-term follow-up are needed to refine patient selection and optimize results.

## Introduction and background

Uterine leiomyomas, commonly referred to as fibroids, are the most prevalent benign smooth muscle tumors of the female reproductive tract, with a lifetime incidence approaching 70% in women of reproductive age. While frequently asymptomatic, fibroids can present with a constellation of clinical symptoms including abnormal uterine bleeding, pelvic pressure, dysmenorrhea, and reproductive dysfunction. Among women seeking fertility, fibroids are found in approximately 5-10% of cases, with submucosal and intramural types more commonly implicated in infertility and adverse pregnancy outcomes. The pathogenesis of fibroids is multifactorial, involving genetic, hormonal, and environmental components. Notably, mutations in the MED12 gene have been identified in a significant proportion of fibroids, underscoring the role of molecular drivers in their development [1]. The clinical impact of fibroids on reproductive potential has garnered increasing attention, particularly as many women delay childbearing into their 30s and 40s when fibroid prevalence is higher.

Surgical management remains the mainstay of treatment for symptomatic fibroids, particularly in women desiring uterine preservation. Myomectomy, the selective surgical excision of fibroids, remains the gold standard for fertility preservation in such cases. Traditionally performed via laparotomy, myomectomy has increasingly shifted toward a minimally invasive approach with the advent of laparoscopic techniques. Laparoscopic myomectomy (LSM) offers several perioperative advantages, including reduced intraoperative blood loss, faster recovery, lower postoperative pain, and decreased adhesion formation, factors that significantly impact future fertility outcomes. These technical and clinical benefits are especially relevant in women seeking to conceive, as a minimally invasive approach may allow for quicker return to fertility efforts and improved uterine function. Advances in surgical instrumentation, imaging guidance, and suturing techniques have further refined the safety and effectiveness of this approach. The review of clinical studies demonstrates consistently high pregnancy rates following LSM, often exceeding 50%, with a significant proportion of women achieving full-term delivery [2].

Despite these promising outcomes, concerns persist regarding the reproductive safety of LSM, particularly the risk of uterine rupture in subsequent pregnancies and deliveries. These concerns have fueled ongoing debate regarding the adequacy of uterine closure during laparoscopy, especially in cases involving multiple or deeply embedded fibroids. In addition, there is significant variability across the literature in terms of study design, patient selection, surgical technique, and follow-up duration, all of which complicate efforts to form definitive conclusions. Consequently, a comprehensive evaluation of pregnancy outcomes following LSM is essential to guide clinical decision-making and optimize patient counseling. This review synthesizes current evidence from observational studies and clinical reports on fertility rates, obstetric outcomes, and maternal complications following LSM, aiming to clarify its role in the reproductive management of women with fibroids.

## Review

Laparoscopic myomectomy (LSM) has become a cornerstone of surgical management in women with symptomatic fibroids who wish to preserve fertility. Compared to traditional open surgery, LSM offers the benefits of reduced intraoperative blood loss, faster recovery, lower postoperative pain, and decreased adhesion formation, factors that significantly impact future fertility outcomes. Advances in surgical instrumentation, imaging guidance, and suturing techniques have further refined the safety and effectiveness of this approach. The review of clinical studies demonstrates consistently high pregnancy rates following LSM, often exceeding 50%, with a significant proportion of women achieving full-term delivery [2].

Fagherazzi et al. reported a 62% pregnancy rate in a cohort of 185 women who underwent LSM, resulting in 115 pregnancies, 111 live births, and no postpartum complications [3]. Among these deliveries, 69 were cesarean sections and 42 were vaginal births. Ribeiro et al. presented outcomes from 28 patients, with 14 total pregnancies, eight cesarean sections and six vaginal births-also without any adverse postpartum events [4]. Koo et al. followed up 523 of 675 patients who underwent LSM and recorded 400 full-term deliveries, including 300 by cesarean section and 100 vaginal births [5]. Only three cases of uterine rupture were reported. Dessolle et al. studied 103 women, 88 of whom had adequate follow-up, and found 44 pregnancies with 36 live births within 12 months of surgery [6]. Kumakiri et al. observed 47 pregnancies in 108 patients, which resulted in 32 live births-19 by vaginal delivery and 13 by cesarean section-managed under vaginal birth after cesarean (VBAC) protocols [7]. No complications were reported beyond a six-month follow-up.

Paul et al. evaluated 217 patients and recorded 141 pregnancies, including 87 cesarean sections, 19 vaginal deliveries, 29 abortions, and 6 ectopic pregnancies [8]. Similarly, Seracchioli et al. reported 158 pregnancies and 106 live births following LSM in 514 women, with 27 vaginal and 79 cesarean deliveries, again without any significant postpartum complications [9]. Dubuisson et al. documented 145 pregnancies and 100 deliveries among 98 patients, including 58 vaginal births and 42 cesarean sections [10]. Three uterine ruptures occurred, with one rupture involving the surgical scar. A second study by Dubuisson et al. included 91 women (mean age 35 ± 4) and documented 51 pregnancies over a two-year follow-up [11]. Bernardi et al. followed 59 women and reported 55 pregnancies and 39 deliveries [12]. Uterine or placental complications were noted in 10-13% of cases over a 73-month follow-up period. Landi et al. assessed 359 patients, reporting 76 pregnancies, including 31 vaginal and 26 cesarean deliveries [13]. The remainder included early trimester miscarriages, one ectopic pregnancy, one molar pregnancy, and one multiple gestation. No major complications were observed.

Rossetti et al. presented 21 pregnancies in 25 patients, with four vaginal and nine cesarean deliveries [14]. Two pregnancies were ongoing at the time of publication, and no complications were noted. Morita et al. reported 15 pregnancies in 30 patients, resulting in five vaginal and 10 cesarean deliveries during a 12-month follow-up [15]. Malzoni et al. included 144 patients, of whom 38 were followed, and reported 34 pregnancies within one year of surgery [16]. Kucera et al. reviewed 351 cases (mean age 33.5 years) with a total of 171 pregnancies [17]. Of these, 46.2% were delivered by cesarean section, and no complications were observed during 12 months of follow-up.

Collectively, these findings underscore that laparoscopic myomectomy is associated with high pregnancy and live birth rates, a relatively low risk of miscarriage or rupture, and excellent maternal outcomes. While cesarean section remains the most frequent mode of delivery, several studies report successful and safe vaginal deliveries, particularly when uterine closure was thorough and surgical technique meticulous. These outcomes reinforce the role of LSM as a highly effective fertility-preserving option for women with uterine fibroids (Table 1).

**Table 1:** Summary of clinical studies reporting pregnancy outcomes following laparoscopic myomectomy

Authors	Cases	Mean Age	Number of Myomas	Number of patients
Fagherazzi et al. [3]	185	19-42	426	115 (111 live births, 69 cesarean section, 42 vaginal birth)-no complications post partum
Ribeiro et al. [4]	28	Not mentioned	28	14 (eight cesarean section, six vaginal birth) -no complications post-partum
Koo et al. [5]	675 (523 follow-up)	Not mentioned	Multiple	400 full term deliveries (300 cesarean section, 100 vaginal births), three uterine rupture
Dessolle et al. [6]	103 (88 follow-up)	36 +/- 2.1	Not mentioned	44 pregnancies, 36 live newborn deliveries, 12-month follow-up
Kumakiri et al. [7]	108	Not mentioned	Not mentioned	47 pregnancies (32 live births, 19 vaginal deliveries, 13 cesarean section - VBAC management) no complications >six month follow up
Paul et al. [8]	217	Not mentioned	Not mentioned	141 pregnancies (19 vaginal deliveries, 87 cesarean section, 29 abortions, six ectopic)
Renato Seracchioli [9]	514	Not mentioned	Not mentioned	158 pregnancies, 106 live births, (27 vaginal deliveries, 79 cesarean section) -no complications post-partum
Dubuisson et al. [10]	98	<45	>98	145 pregnancies, 100 deliveries (58 vaginal, 42 cesarian deliveries), three uterine ruptures (one on the scar)
Dubuisson et al. [11]	91	35 +/- 4	>91	51 pregnancies, two-year follow-up
Bernardi et al. [12]	59	23-42	Not mentioned	55 pregnancies, 39 deliveries, (uterine, placental complications 10-13 %), 73-month follow-up
Landi et al. [13]	359	Not mentioned	Not mentioned	76 pregnancies, (one ectopic, one multiple, 12 1^st^ trimester miscarriages, one hydatiform) 31 vaginal, 26 cesarian deliveries no complications
Rossetti et al. [14]	25	Not mentioned	Not mentioned	21 pregnancies (four vaginal, nine cesarian deliveries) two ongoing – no complications
Morita et al. [15]	30	Not mentioned	>30	15 pregnancies, (five vaginal, 10 cesarian deliveries) 12 months follow-up – no complications
Malzoni et al. [16]	144 (38 followed up)	Not mentioned	144	34 pregnancies, one-year follow-up
Kucera et al. [17]	351	33,5 (average)	643	171 pregnancies, (46,2% cesarian deliveries) 12-month follow-up – no complications

Comparison with open myomectomy

Open myomectomy (OM), traditionally performed via laparotomy, has long been the standard surgical approach for the removal of uterine fibroids, especially in cases involving large, numerous, or deeply embedded myomas. However, with the advancement of minimally invasive techniques, laparoscopic myomectomy (LSM) has become increasingly favored in women who wish to preserve fertility. The adoption of LSM reflects not only its technical evolution but also growing evidence of its comparable reproductive outcomes and superior perioperative profile.

The studies included in this review collectively suggest that LSM achieves fertility outcomes similar to those observed following OM. For example, pregnancy rates reported by Fagherazzi et al. [3], Ribeiro et al. [4], and Koo et al. [5] after LSM are comparable to those historically associated with open procedures. Specifically, Koo et al. [5] documented 400 full-term deliveries in 523 patients, a number that demonstrates the potential of LSM to match or even exceed the success rates of open approaches in appropriately selected patients.

In terms of surgical morbidity, LSM offers several advantages. Reduced intraoperative blood loss, shorter hospital stay, faster postoperative recovery, and decreased adhesion formation are consistently reported across studies. These benefits are especially relevant in the context of fertility preservation, as postoperative adhesions can compromise tubal function and uterine mobility, thereby impairing natural conception. Dessolle et al. [6] and Kumakiri et al. [7] both described uncomplicated postoperative courses in their cohorts, with high pregnancy and live birth rates, suggesting that the minimally invasive nature of LSM may contribute to improved overall fertility prospects.

Nevertheless, one of the key controversies surrounding LSM in comparison to OM involves the risk of uterine rupture in subsequent pregnancies. The tactile feedback and broader exposure during OM may theoretically allow for more secure multilayer closure of the myometrium. However, multiple studies, including those by Seracchioli et al. [9], Paul et al. [8], and Landi et al. [13], have shown that when laparoscopic suturing is performed by experienced surgeons using appropriate multilayer techniques, uterine integrity is preserved and reproductive outcomes are not compromised.

Dubuisson et al. did report three cases of uterine rupture, one of which occurred at the myomectomy scar, reinforcing the importance of surgical technique [10]. However, these events were rare and did not lead to significant maternal or neonatal morbidity. Similarly, Kucera et al. documented 171 pregnancies with no complications in a cohort of 351 women, supporting the long-term safety of laparoscopic approaches when performed with proper technique and postoperative counseling [17].

In summary, LSM yields outcomes comparable to OM in terms of pregnancy and live birth rates, with the added benefits of minimally invasive surgery. While uterine rupture remains a concern, particularly in patients with large or multiple fibroids, the overall risk appears low when closure is meticulous and the interval between surgery and conception is appropriate. For women with fibroids who desire future fertility, LSM should be considered a first-line surgical option in the hands of experienced surgeons.

Uterine rupture and mode of delivery

One of the most debated concerns following laparoscopic myomectomy (LSM) is the risk of uterine rupture during pregnancy or labor, particularly in patients undergoing trial of labor after surgery. While rare, uterine rupture can have serious maternal and fetal consequences, and it is often cited as a reason to favor cesarean delivery in women with a history of myomectomy. However, the evidence from the reviewed studies suggests that the actual incidence of rupture after LSM is low, especially when surgery is performed with proper multilayer uterine closure and adequate healing time is observed before conception.

In the largest available series, Koo et al. reported only three cases of uterine rupture among 400 full-term deliveries, indicating a rupture rate of less than 1% [5]. Importantly, these cases did not result in significant adverse outcomes. Similarly, Dubuisson et al. noted three uterine ruptures among 100 deliveries, with only one rupture occurring at the surgical scar site [10]. In both reports, uterine rupture was more likely when pregnancies occurred early after surgery or when uterine repair may have been suboptimal.

In contrast, the majority of studies, such as those by Fagherazzi et al. [3], Ribeiro et al. [4], Seracchioli et al. [9], and Kucera et al. [17], reported no cases of uterine rupture, even in patients who delivered vaginally. These findings suggest that the risk is not inherently higher after LSM compared to open myomectomy, but rather depends on surgical technique, fibroid location, and the structural integrity of uterine repair. Kumakiri et al. [7] specifically reported 19 successful vaginal deliveries following LSM without any complications, using a structured approach to VBAC-style monitoring.

Cesarean section remains the predominant mode of delivery following LSM in the majority of reviewed studies. For example, Paul et al. [8] reported 87 cesarean deliveries out of 141 pregnancies, while Seracchioli et al. [9] observed 79 cesarean sections among 106 live births. This tendency likely reflects a combination of caution on the part of obstetricians, lack of consensus guidelines, and medico-legal concerns. However, data from Rossetti et al. [14], Morita et al. [15], and Landi et al. [13] document successful and uncomplicated vaginal deliveries after LSM, suggesting that a universal recommendation for cesarean section may be overly conservative.

The decision regarding delivery mode should be individualized based on factors such as the size and location of the fibroids removed, the depth of uterine incisions, the method of closure, and the length of the interpregnancy interval. In patients with superficial fibroids, single uterine incisions, and adequate healing, vaginal delivery may be a reasonable and safe option, provided that close intrapartum monitoring is available. Overall, the reviewed evidence supports the conclusion that uterine rupture after LSM is an uncommon event and that the mode of delivery should not be dictated solely by the surgical approach, but rather by a comprehensive obstetric risk assessment.

Limitations of the current literature

While the collective findings support the efficacy and safety of laparoscopic myomectomy (LSM) in women seeking fertility preservation, several limitations across the reviewed literature must be acknowledged. These limitations reflect both the methodological challenges of studying reproductive outcomes and the variability in surgical practice among centers. A primary limitation is the predominance of retrospective cohort studies and case series, which are inherently subject to selection bias, confounding, and lack of control groups. For example, studies such as those by Fagherazzi et al. [3], Ribeiro et al. [4], and Morita et al. [15] report high pregnancy and live birth rates; however, they often include patients with favorable baseline fertility potential, limiting generalizability. Few of the included studies employed prospective designs, and even fewer offered randomized comparisons with open myomectomy or non-surgical management.

Another important issue is the lack of standardization in surgical technique. Critical variables such as the number of fibroids removed, the depth of uterine incisions, the use of energy devices, and the method and number of suture layers used are inconsistently reported or completely omitted. For instance, while Koo et al. [5] and Seracchioli et al. [9] report excellent outcomes, their methods of uterine closure are not detailed enough to allow for reproducibility or meaningful subgroup analysis. The follow-up duration also varies considerably between studies, with many reporting outcomes over 6 to 12 months. Only a few studies, such as those by Bernardi et al. [12] and Landi et al. [13], provide extended follow-up beyond two years. This limits the ability to assess long-term fertility potential, the durability of surgical outcomes, and late complications such as uterine rupture during labor or abnormal placentation in subsequent pregnancies. Moreover, reporting of adverse reproductive outcomes, such as miscarriage, ectopic pregnancy, and uterine rupture, is inconsistent. Some studies, such as those by Paul et al. [8] and Dubuisson et al. [10], offer detailed accounts of such events, while others do not specify whether complications occurred. This raises the possibility of underreporting or publication bias, especially in smaller series or single-center experiences.

Another limitation is the absence of standardized definitions for surgical success, reproductive endpoints, and patient inclusion criteria. This heterogeneity makes it difficult to pool data or conduct meta-analyses that could more definitively quantify the reproductive benefits and risks of LSM. Finally, many studies fail to control for important confounding variables such as patient age, prior fertility treatments, fibroid subtype, and coexisting pelvic pathologies (e.g., endometriosis), all of which can independently affect reproductive outcomes. For example, Kumakiri et al. [7] and Kucera et al. [17] report high conception rates, but do not stratify patients by fibroid size or location, which could significantly influence prognosis.

In summary, while current evidence supports the role of LSM in fertility preservation, the lack of standardized surgical and reporting protocols, short follow-up periods, and inconsistent documentation of complications highlights the need for well-designed, multicenter prospective trials. These studies should aim to evaluate surgical technique, uterine healing, timing of conception, and delivery mode in a standardized and stratified manner to better inform clinical practice.

## Conclusions

Laparoscopic myomectomy (LSM) represents a safe, effective, and fertility-preserving surgical option for women with symptomatic uterine fibroids who wish to conceive. The reviewed literature demonstrates high pregnancy and live birth rates following LSM, with a low incidence of serious complications such as uterine rupture. These outcomes are comparable to, and in some cases exceed, those observed with open myomectomy, particularly when surgery is performed by experienced laparoscopic surgeons using appropriate multilayer uterine closure techniques.

While cesarean section remains the most common mode of delivery after LSM, several studies confirm the feasibility and safety of vaginal delivery in selected patients. The choice of delivery route should therefore be individualized, based on surgical findings, fibroid characteristics, and obstetric evaluation, rather than determined solely by surgical approach.

In conclusion, laparoscopic myomectomy offers substantial reproductive benefits in appropriately selected patients and should be considered a first-line surgical approach when performed by experienced hands. Comprehensive patient counseling, meticulous surgical technique, and structured postoperative monitoring are essential to ensure favorable outcomes in this unique patient population.
